# One million global catheters PIVC worldwide prevalence study: building alliances to put vascular access device management and infection prevention on the world stage from Australia to the Democratic Republic of Congo

**DOI:** 10.1186/2047-2994-4-S1-P204

**Published:** 2015-06-16

**Authors:** EK Kabululu, G Ray-Barruel, E Alexandrou, CM Rickard

**Affiliations:** 1 Equipe Médicale des volontaires, Beni Nord KIVU, Congo, The Democratic Republic of the; 2 OMG PIVC Study Investigators, Nathan; 3 OMG PIVC Study Investigators, Liverpool, Australia

## Background

Despite the existence of numerous guidelines for peripheral intravenous catheter (PIVC) insertion and care, outcomes for peripheral catheters are reportedly sub-optimal. Furthermore, little is known about PIVC outcomes in developing nations. Optimising infection control of PIVCs is a unique challenge for developing and least developed countries. Following devastating events in 2002, the Centre Médical Evangélique de Nyakunde in Democratic Republic of Congo is slowly rebuilding hospital capacity and tackling the challenges of infection control in difficult conditions.

## Aim

To conduct a global prevalence study to analyse current practices of PIVC management, identify areas for improvement in catheter care and infection prevention, and support hospital staff across the world to build research capacity in vascular access device care.

## Methods

Interest in the study was spread via professional networks, conferences, newsletters, industry partners, social media, and global research networks. Local investigators assumed the role of study champion, promoting the study in their own country. Participants included staff nurses and physicians, infection control and vascular access clinicians, nurse educators, and nursing and medical researchers. Multiple language options were provided. On a given day, decided by each organisation, consenting hospital patients permitted the details of their PIVC to be collected for the study. No identifying patient data was collected.

## Results

More than 750 hospitals in 65 countries participated in the study from June 2014 until April 2015. The Centre Médical Evangélique de Nyakunde will present their reasons for participation and unique challenges faced during the experience of participating in this global study. Key issues identified by this central African hospital included inadequate internet connection, lack of knowledge of best practice in PIVC insertion and management, and subsequent waste of valuable resources.

## Discussion

Enthusiasm for this global prevalence study has led to the development of cross-cultural nursing and medical research partnerships in over 65 countries. With networks and research pathways now established, opportunities abound for further collaborative research to improve care of vascular access devices and prevent catheter-related bloodstream infection. It is hoped that twinning opportunities for Centre Médical Evangélique de Nyakunde will arise as a result of participation in this research.

## Disclosure of interest

None declared.

